# Safety of epoietin beta-quinine drug combination in children with cerebral malaria in Mali

**DOI:** 10.1186/1475-2875-8-169

**Published:** 2009-07-24

**Authors:** Stéphane Picot, Anne-Lise Bienvenu, Salimata Konate, Sibiri Sissoko, Abdoulaye Barry, Elisabeth Diarra, Karidiatou Bamba, Abdoulaye Djimdé, Ogobara K Doumbo

**Affiliations:** 1 Malaria Research Unit, EA 4170, University Lyon 1, Faculty of Medicine, 8 avenue Rockefeller, 69373 Lyon, France; 2 Paludisme, parasites du sang et mycologie médicale, Hospices Civils de Lyon, Lyon, France; 3 Malaria Research and training Center, Department of Epidemiology of Parasitic Diseases, Faculty of Medicine, Pharmacy and Dentistry, University of Bamako, Mali

## Abstract

**Background:**

Cerebral malaria carries an unacceptable case fatality rate in children despite timely and adequate chemotherapy. To improve the survival rate, adjunctive therapies previously tested mainly focused on the modulation of the inflammatory response, without definitive effect in humans. In this context, a new adjunctive strategy using a neuroprotective drug: erythropoietin (epoietin-beta, Epo) was proposed.

**Methods:**

An open-labelled study including cerebral malaria children (Blantyre coma score below 3) was conducted in Mali. The objective was to assess the short-term safety (seven days) of erythropoietin at high doses (1,500 U/kg/day during three days) combined to quinine.

**Results:**

35 patients with unrousable coma were included in the study. None of expected side effects of erythropoietin were observed during the seven days follow-up. No significant increase in the case fatality rate (7/35 patients) was observed compared to other studies with mortality rates ranging from 16 to 22% in similar endemic areas.

**Conclusion:**

These data provide the first evidence of the short-term safety of erythropoietin at high doses combined to quinine. A multicentre study is needed to assess the potential of Epo as an adjunctive therapy to increase the survival during cerebral malaria.

**Clinical registration number:**

ClinicalTrials.gov ID: NCT00697164

## Background

Malaria is one of the most common life-threatening diseases in sub-Sahara Africa with a dramatic impact on public health: more than one million deaths each year. Cerebral malaria (CM) carries unacceptable case fatality rate in children despite timely and adequate chemotherapy. The SEAQUAMAT group provided the first evidence of artesunate superiority (mortality rate: 15%) compared to quinine (mortality rate: 22%) during severe falciparum malaria in adults [1]. However, available artemisinin-based combination therapy (ACT) is not associated with a complete recovery in case of severe malaria, suggesting a potential role for adjunctive therapies in the early phase of the disease. Most adjunctive therapies tested in human cerebral malaria were focussed on the modulation of the inflammatory response [2]. However, none of the trial using adjunctive therapies from data obtained in animal models provides an increase in survival or an improvement of patient outcome [3-5]. It was highlighted that most of these studies were underpowered [6]. In some cases, these adjunctive drugs were responsible for deleterious effects and trials were suspended [7]. A new opportunity was opened when the beneficial effect of neuro-protective drugs, including erythropoietin (epoietin-beta, Epo), was demonstrated both in animals [8,9] and humans during stroke [10]. The neuro-protective effect is mediated through the inhibition of cell death in the ischemic penumbra surroundings the tissue injury [11]. There is now a growing evidence for this concept of cell protection during acute ischemic diseases [12,13].

Epo has recently been considered in a new light as a tissue-protective cytokine. Its neuro-protective properties have attracted considerable attention: Epo and its receptor (EpoR) are part of a highly potent endogenous neuro-protective system in the brain [14]. Similar to the mechanism of action shown for cells in the haematopoietic system, Epo acts on cells of the nervous system via binding to its specific receptor EpoR. Dimerizing of EpoR leads to autophosphorylation of the receptor associated Janus tyrosine kinase 2 and activation of distal signal transduction cascade: phosphatidylinositol-3-kinase (PI3-K), akt protein kinase, RAS mitogen-activated protein kinases, signal transducers and activators of transcription-5 (STAT-5) and NF-kB-dependant transcription [15]. The underlying mechanisms are hypothesized to be multifactorial. Epo may antagonize the cytotoxic effect of glutamate, increase expression of antioxidant enzymes, reduce nitric oxide mediated formation of radicals, normalize cerebral blood flow, influence neurotransmitter release and promote neuroangiogenesis [16]. All these mechanisms are contributing to attenuation of apoptosis [8,9] and reduction of brain inflammation [9].

The effect of neuro-protective therapies was not previously investigated during malaria in humans, although the manifestations of cerebral malaria partly share features with neurological stroke, neuron damage related to ischaemia-reperfusion stress or acute non-specific neurological disorders. From experimental malaria, it was speculated that the progression from cerebral malaria to death could be counteracted by Epo high doses at the onset of clinical phase of the disease [17].

While the safety of Epo in chronic diseases is known, using high doses for a short period of time is a major issue to be addressed in severe cerebral malaria patients. Indeed, in September 2008, FDA has been made aware of preliminary safety concerns from a clinical trial conducted in Germany investigating the use of epoietin-alfa high doses (40,000 units daily for three days) to treat acute ischemic stroke, while investigators considered this as premature alert. Early in 2009 another clinical trial using epoietin-alfa high doses (1,000 unit/kg/day for seven days) in extremely premature infants to prevent/attenuate brain injury was also FDA hold. In both cases, epoeitin-alfa was used and FDA did not communicate their final conclusions and recommendations to the public. The *in vivo/in vitro *bioactivity ratio is more than 20% higher for epoietin-beta than for epoietin-alpha, because epoietin-beta is less sialylated than epoietin-alpha [18,19]. Desialylation of Epo is known to reduce the plasma survival of Epo and the biological effect and to increase the affinity of Epo for its receptors. To obtain the appearance of exogenous Epo in the cerebrospinal fluid and the neuro-protective effect, Epo doses higher than those used for anaemia treatment are needed [20].

To document the safety of epoietin-beta high doses (1500 U/Kg) at day 7 post-admission, combined to quinine, the local recommended drug for cerebral malaria, a clinical trial in Bamako (Mali) including children admitted with highly critical stage cerebral malaria (Blantyre coma score <3) was conducted. The safety was defined as the lack of severe adverse effects attributable to Epo.

## Methods

### Study design

This was an open-labelled study conducted in Gabriel Toure Hospital, one of the national referral hospitals of Bamako, Mali. Ethical approval was obtained from the Institutional Review Board (IRB) of the Faculty of Medicine, Pharmacy and Dentistry of Bamako, and from the board of trustees of Roche Foundation for Anaemia Research (RoFAR). The trial was registered (NCT00697164, clinicaltrials.gov). The funders had no role in the study design, data collection, analysis, decision to publish or preparation of the manuscript. Written informed consent was obtained from parents or legal guardians of patients prior to enrolment.

Children between six months and 14 years old, presenting cerebral malaria with a Blantyre coma score <3 due to *Plasmodium falciparum *infection (confirmed by blood thick smears microscopical examination), were included in the study after consent obtained from parents or legal guardians. All other categories of severe malaria, including severe anaemia (defined by haemoglobin levels below 5 g/dL), respiratory distress and acute renal failure were excluded. Children with other neurological disorder (epilepsy, cerebral palsy), known sickle cell disease and inter-current infections were excluded.

Cerebral malaria patients received anti-malarial treatment according to the recommendation of Mali National Malaria Control Programme. They received quinine intravenously 20 mg salt/kg bw on admission then 10 mg salt/kg bw every eight hours from day 1 to day 3. Then, they received artesunate-amodiaquine combination (4 mg/kg/day and 10 mg/kg/day, respectively) from day 4 to day 6. Adjunctive therapy was based on intravenous infusion of 1500 U/kg/day rHuEPO (epoietin-beta, Neorecormon^®^) from admission to day 3 (three doses). Considering the very low risk of side effects with a short-term rHuEPO treatment, 35 patients were included, which is within the range of a generally accepted sample size for this purpose.

The primary end point was survival of patients by day 7 post-admission. Due to the lack of follow-up information after discharge from hospital, and to the impossibility for patients to come back to outpatient clinic for economic reasons, we could not ascertain the long-term outcome patients who survived.

Secondary end points were i) incidence of adverse effects, ii) incidence of abnormal laboratory values, iii) rate of neurological disorders evaluated by clinical examination and Blantyre coma score.

An independent local Medical Monitor and an external Data and Safety Monitoring Committee, composed by four members, were designed and evaluated adverse events and other safety data. Reports of cases were regularly sent to the data monitoring committee. The case causalities were assessed using the standardized WHO-UMC system by DSMC members. All the investigators completed IRB certificate for clinical trial and passed the evaluation from the Collaborative Institutional Training Initiative (CITI, University of Miami and Fred Hutchinson Cancer Research Center). The study adhered to the international conference on Harmonization Guidelines for Good Clinical Practices and was conducted in accordance with the Declaration of Helsinki.

### Trial procedures

The following data were collected from all enrolees on a standardized case report form. Demographic details (date of birth or age, gender, weight, ethnic group, address), history of present illness (delay before admission, convulsions and coma prior to admission, date and time of admission, anti-malarial treatment before admission), physical and biological findings were gathered on admission and every day during the follow-up. Standard operating procedures were developed, applied to all patients and were strictly respected. The clinical team was trained in operating and maintaining the instruments. Biological quality controls for all the biological parameters were performed once a week. Biological abnormalities were double-checked. Regular meetings were held to ensure that data quality was maintained.

Clinical and biological monitoring was in agreement with the WHO guidelines for cerebral malaria [21]. Temperature, Blantyre coma score, respiratory status, seizure, blood pressure, cardiac frequency, jaundice, dehydratation were recorded on admission and every four hours from day 1 to day 3, then every eight hours by the end-point.

Parasitaemia (Giemsa-stained thick smears), haemoglobin level (Hemocue Hb 201+^®^, Hemocue), blood saturation (Fingertip 300D^®^, RDSM), blood lactate (Lactate Pro^®^, FaCTCanada), blood gas analysis (pHOx Basic, Novabiomedical), diuresis, haemoglobinuria (dipstick urinalysis), were recorded daily. Blood glucose levels (Accu-Check Go^®^, Roche diagnostic) were recorded every eight hours for the first two days, then daily. Creatininaemia and alanine aminotransferase assays were referred to local reference laboratory.

Continuing supportive care was provided according to standard recommendation [21] and patient were treated with the highest level of care available on site. However, neither oxygen nor mechanical ventilation was available in this paediatric emergency ward. Repetitive seizures were treated by diazepam. Hypoglycaemia, anaemia and acidosis were treated empirically.

### Statistical analysis

This study was the first part of a wider-sized project aimed to evaluate the proof-of-concept of epoietin-beta as adjunctive therapy during cerebral malaria. At this step, the study was designed to demonstrate the short-term safety of Epo high doses in endemic area low-income country with poor medical capabilities. The effects of high-dose recombinant human epoietin-beta for 35 consecutive patients who have been admitted with a diagnosis of highly severe cerebral malaria were studied. Epo was injected intravenously shortly after admission and subsequently during 2 days. Safety analysis was undertaken for patients who were assigned to receive at least one dose of study drug.

Two different people performed data entries. Database discrepancies were visually checked against source documents and resolved by consensus. Assessment of demographics, clinical and laboratory variables were based on descriptive statistics including mean, standard deviation, medians, and proportions. Mean values of the vital sign and biological parameters were compared using a Student *t*-test for continuous variables with a normal distribution. Baseline characteristics were assessed with Fisher's exact test for categorical variables. *P*-values were based on two-sided significant level of 5%.

## Results

### Baseline characteristics

Overall, 35 patients were included in the study between October 2007 and September 2008. 28 patients received full treatment including three doses of Epo at 1,500 UI/kg combined with quinine and completed follow-up according to the protocol. Seven patients died before the end of the treatment and received only one or two doses of treatment.

Table 1 shows baseline patients characteristics. At time of admission, a similar number of patients presented a Blantyre coma score of 1 (17 patients) and 2 (18 patients). The population showed normally distributed ages with a median of 6 years (range: 2–12). Most of the patients were febrile (> 37.4°C) at admission except 7 (20%). No information was available concerning the use of antipyretic drugs self-medication. The mean duration of fever before reference to the hospital was 75 ± 32 hours (range: 12–148). Median parasitaemia value was 7,100 asexual blood forms of mono-specific *P. falciparum *detected on thick blood films, and PCR confirmed from paper blood spots. All patients presented haemoglobin levels on admission from 7.0 to 12.4 g/dL (median: 9.3 g/dL). None of the patients presented severe hypoglycaemia except two, who were treated before admission with a three-day course of quinine (13 mg/kg/day) or high dose chloroquine (700 mg total dose) given by a primary health care officer.

**Table 1:** Baseline and disease characteristics in the included population

	**Demographics and Disease Characteristics**			**Statistical analysis**
	Intention to treat (n = 35)	Surviving patients (n = 28)	Deceased patients (n = 7)	*p*-value surviving vs deceased

**Baseline Characteristics:**	**Mean ± standard deviation (range)**			**Student *t*-test**
	
Sex ratio (M/F)	1.7 (22/13)	2.1 (19/9)	0.75 (3/4)	
Age, years	6.3 ± 2.5 (2–12)	6.2 ± 2.7 (2–12)	6.7 ± 2.4 (3–9)	.60
Weight, kg	19.5 ± 6.9 (10–35)	19.7 ± 7.5 (10–35)	18.9 ± 6.0 (11–26)	.62
Temperature,°C	38.6 ± 1.3 (36.2–40.6)	38.7 ± 1.4 (36.2–40.6)	37.8 ± 1.0 (36.6–39.6)	.08
	
**Clinic day 1:**	**Patient ratio (%)**			**Fisher *F*-test**
	
Blantyre score = 1	17/35 (49%)	12/28 (43%)	5/7 (71%)	.32
Blantyre score = 2	18/35 (51%)	16/28 (57%)	2/7 (29%)	
≥ 1 seizure	23/35 (66%)	17/28 (61%)	6/7 (85%)	.16
Deep breathing	7/35 (20%)	4/28 (14%)	3/7 (42%)	.06
Haemoglobinuria	7/35 (20%)	6/28 (21%)	1/7 (14%)	1
Jaundice	4/35 (11%)	4/28 (14%)	0/7 (0%)	.55
	
**Biological values day 1**	**Mean ± standard deviation (range)**			**Student *t*-test**
	
SaO2, %	91.0 ± 8.7 (73–98)	89.8 ± 9.4 (73–98)	95.1 ± 3.5 (88.7–98)	.10
asexual	43,000 ± 62,000	45,000 ± 46,000	37,000 ± 54,000	.85
*P. falciparum*/μL	(200–250,000)	(200–250,000)	(625–140,000)	
Blood pH	7.29 ± 0.1 (7.1–7.43)	7.3 ± 0.1 (7.2–7.4)	7.3 ± 0.2 (7.1–7.4)	.72
Blood Lactates, mmol/L	6.0 ± 3.6 (1.4–15.4)	5.8 ± 3.3 (1.4–15.4)	6.3 ± 3.6 (2–11.7)	.78
Haemoglobin, g/dL	9.4 ± 1.4 (7.0–12.4)	9.6 ± 1.4 (7.0–12.4)	9.0 ± 1.2 (7.1–10.3)	.32
Plasma glucose, mmol/L	6.4 ± 3.6 (0.8–19.4)	6.3 ± 2.3 (1.1–11.3)	7.8 ± 6.1 (1.25–19.4)	.54
Plasma creatinin, μmol/L	57 ± 28 (21–130)	58.4 ± 26.0 (29–130)	36.7 ± 14.8 (21–60)	.02
ALAT, IU/L	87 ± 129 (7–600)	100 ± 150(7–600)	47 ± 18 (22–71)	.11

Clinical and biological data were gathered at the time of admission. Included patients were stratified according to the primary end-point outcome (surviving or died patients on day six post admission). Student *t*-test or Fisher *F*-test were used to compare the two groups when appropriate.

### Clinical outcomes

For the primary intention-to-treat analysis (35 patients), there were no significant differences in demographic (Student *t*-test) or disease characteristics (Fisher *F*-test) between the surviving and the deceased patients (table 1). Seven patients died during the early stages of the hospitalization (all before 48 hours after admission). The circumstances of these deaths were as followed:

- One child (9 years old, 26 kg) was included and treated at admission with Epo, quinine, diazepam intramuscularly and phenobarbital for repeated seizures, but without parasite blood stage documentation. He was later classified as protocol violation. He died one hour after admission without aetiologic diagnosis.

- One child (3 years old, 11 kg) received a total dose of 700 mg of chloroquine the week before admission and was admitted with a Blantyre score at 1, repeated seizures, profound hypoglycaemia and respiratory pauses. He received at admission quinine and Epo, plus 5 mg diazepam and 40 mg phenobarbital, and died three hours later.

- One child (7 years old, 20 kg) was admitted with repeated seizures that were not terminated by initial injection of diazepam (10 mg) intramuscularly. He received successively two more diazepam injections and 120 mg phenobarbital during the first 30 hours, while he presented respiratory pauses. Mechanical ventilation and oxygen were not available and the patient died 44 hours post-admission after respiratory distress. He received two doses of Epo.

- One child (4 years old, 13 kg) was admitted with a Blantyre coma score of 2, with seizures and respiratory distress. Clinical improvement was observed after two doses of quinine and Epo. The children received enteral nutrition prepared by his family without septic care at 20, 24 and 28 hours post-admission. He suddenly presented vomiting during four hours, hypotension, mydriasis and respiratory arrest at 32 hours post-admission. No bacterial culture could be obtained during the time of the lethal shock due to the lack of these facilities during the night.

- One child (8 years old, 21 kg) was admitted with deep acidosis (blood pH = 7.1, controlled) not taken into account by medical team until 24 hours when the blood pH was measured at 7.0. He received Ringer 500 mL after 24 and 48 hours without biological and clinical improvement and died after 51 hours.

- Two children (aged 7 & 9) died immediately and two hours post-admission, respectively, the first deceased child was nearly impossible to treat using intravenous route due to severe hypotension and the second with high blood lactate level (11.7 mmol/L). They received quinine and Epo treatment at admission, but no resuscitation was possible.

The five first of these deaths were classified by the DMC as unlikely due to Epo according to the WHO-UMC system. The others two children were classified as non-evaluable.

28/35 patients survived at day 7 post-admission (80%). Although the study was not designed to detect a treatment effect on the time to recovery, the Blantyre coma score increased from stages 1–2 to stages 4–5 in less than 48 hours.

### Short-term safety

Epo is a safe drug used for years to treat chronic anaemia caused by renal failure or cancer. Here much higher doses of that drug were used during a very short period of time. Side effects suspected to be due to high doses were monitored.

None of the known side effects of EPO including increase in blood pressure [22], and venous thrombosis due to excessive erythropoeisis, were observed during the 7 days follow-up [23]. Among the surviving patients, no increase of haemoglobin blood level was observed during the time of Epo treatment, between admission (mean = 9.4 ± 1.4 g/dL), day 3 (mean = 9.0 ± 2.5 g/dL) and day 7 (mean = 8.3 ± 1.8 g/dL). The decrease in haemoglobin level between the mean values at admission and day 3 was not significant (Student *t*-test > 0.05), while it became significant at day 7 (Student *t*-test = 0.03).

## Discussion

Child mortality from malaria is still unacceptable in sub-Saharan Africa, while ACT was deployed in many endemic areas. These drugs are quite effective in clearing the blood stage parasites within 48 to 72 hours [24]. Most deaths occur within 24 hours of admission to hospital meaning before anti-malarial drugs effect [25]. This well-known observation provides a therapeutic window for adjunctive therapies administration, particularly those targeted the host cells protection. Adjunctive therapies have been tested in humans but none clearly showed to be beneficial [26]. Most of these trials were designed to block the downstream effects that occur as a result of *P. falciparum *infection or to resuscitate the patients using aggressive fluid therapy [27]. More recently, the combination of erythropoietin high doses with artesunate showed a reduced delay before clinical recovery and a 50% mortality decrease in mice [28].

The haematopoietic growth factor Epo has been used as a drug for the treatment of anaemia for almost two decades. Since Epo and Epo-Receptor are expressed in several organs (including kidney, brain, heart, muscle, endothelial cells), Epo function is not limited to the haematopoietic system [29]. Systemic administration of exogenous Epo regulates cerebral blood flow, protects endothelial cells against oxydative stress and reduces inflammation by inhibiting pro-inflammatory cytokines [30]. These effects are partly due to the inhibition of cell apoptosis and could explain the tissue protective potential of Epo [31]. Epo was tested in a proof-of-concept trial during stroke: it was shown to be well tolerated and associated with an improvement in clinical outcome after one month [10]. Epo exhibits its high non-specific potential in various other pathological conditions including renal failure and schizophrenia [31].

Since the pathological mechanisms involved in ischaemia share features with cerebral malaria, and none of the anti-malarial drug is effective to control the progression of the disease in the first hours of treatment, there was an opportunity for the use of Epo as an adjunctive therapy in the early stage of cerebral malaria [32].

Children (median age: 6) referred to hospital after up to 6 days of fever were included. These children were living in urban or peri-urban settings with mesoendemic transmission level. The overall sex ratio was 1.7 (male/female) compared to 0.75 (3 males/4 females) for patients who died at hospital.

Most of the patients received incomplete treatment (anti-malarial drugs locally available) in primary health care centres and were referred to hospital in the absence of clinical improvement, leading to the late stages of unrousable coma observed at admission. Among the patients included, there was a similar number of patients showing a Blantyre coma score of 1 and 2 (17 and 18 respectively). The aim of this study was to investigate the safety of Epo during cerebral malaria. Patients suspected to present respiratory distress or renal failure before admission could lead to a bias in the interpretation of the results. While severe malaria is often a combination of severe anaemia and cerebral malaria, patients with a significant decrease in haemoglobin levels were not included to avoid any interference in the data analysis with the potential effect of Epo on erythropoiesis. However, it has to be remembered that authors [10] demonstrated in a pilot study that 3 days of Epo at high doses did not increase haemoglobin to levels exceeding the normal range, excluding the need to use non-haematopoietic Epo derivatives such as carbamylated erythropoietin in that condition. This was confirmed here since a significant decrease in haemoglobin level was observed at day 7, as expected during anti-malarial treatment. Considering the low level of haemoglobin in these children at discharge (mean = 8.3 ± 1.8 g/dL), very common in endemic areas, it could be speculated that a delayed increase in erythropoiesis will not lead to abnormal or pathological red blood cells mass. None of the patients showed blood mass increase, vascular thrombosis or hypertension along the 7 days follow-up. Among the patients who survived, the neurological clinical improvement was observed as expected in the first two days of treatment.

Seven patients/35 died during the trial. Five were classified as unlikely due to Epo high doses by the DSMC and none of these deaths should be attributed to Epo. Among them, two patients received only one Epo dose and were admitted with extremely severe symptoms and deep coma. In the emergency situation, patients admitted with low grade coma score were included and received Epo treatment. Some patients were included despite the presence of respiratory distress. Nevertheless, it was decided to present these information in intention-to-treat protocol to avoid any bias in data analysis.

It is well recognize that good supportive care and management of complications are essential in the treatment of severe malaria [6]. Paediatric resuscitation presents a great challenge for health workers who are faced with inadequate facilities and often advanced disease in children. Deficiency in breath arrest management is one of the major factors that could influence the outcome of the resuscitation. Survival also depends on underlying diseases and the time elapsing between arrest and the initiation of the resuscitation. Broader provision of ventilation facilities and early identification of cases that need intubation and ventilation may improve the prognosis of cardiopulmonary arrest. However, in many, if not mostly paediatric wards in endemic areas hospitals, staffing levels, clinical supplies and laboratory capacities are limited [33]. The key question is whether clinical studies conducted in resource-poor settings could help to improve the therapeutic management of patients with cerebral malaria, or to enhance only the capacity of local team involved in these studies. Here, it could be speculated that the capacity building needed to conduct this trial was necessary and sufficient to demonstrate the safety of Epo high doses in the resuscitation of children admitted with unrousable coma and poor prognosis. It could be deplored that several patients died because standard protocols used in western intensive care units were not available. This field reality did not preclude the value of the demonstration of Epo high doses safety, the main objective of this study.

The comparison of case fatality rates with other cerebral malaria studies in different settings are always controversial. However, considering the severity of the disease at admission and lack of standard supportive care for children included in this study, the observed mortality rate of 20% could be analysed in the light of the case fatality rate of 16.8% in Kenya [34]; 19,3% in SMAC network [35], 22% in Kenya [36]; 27% expected from five studies in comparable endemic areas [26]. A recent study conducted in the same ward showed a 16% case fatality rate for patients admitted with cerebral malaria whatever the coma score was [37]. Authors demonstrated that the lowest the Blantyre score, the highest the mortality.

## Conclusion

Taken altogether, these data demonstrated that the short term safety (seven days) of Epo high doses administered during three days was obtained in children presenting extremely severe cerebral malaria. Before these findings can be considered as a clinical directive, the long-term safety of Epo need to be further evaluated in larger-scale, longer-term clinical studies. A randomized, double-blinded, multi-centre trial will provide definitive information on the potential of Epo as an adjunctive therapy in humans and the effect of Epo plus anti-malarial drug combination on CM case fatality rate [6]. The control of apoptosis in brain during severe ischaemia-like injury could be a new therapeutic opportunity in the future. Here, in agreement with the Mali National Malaria Control Programme, cerebral malaria patients were treated using quinine during the first three days. It is of interest to test the beneficial effect of Epo combined with a more potent ACT.

## Ethics committee approval

Ethical approval was obtained from the Institutional Review Board (IRB) of the faculty of Medicine, Pharmacy and Dentistry of Bamako, and from the board of trustees of Roche Foundation for Anemia Research (RoFAR). The trial was registered (NCT00697164, clinicaltrials.gov).

## Competing interests

The authors declare that they have no competing interests.

## Authors' contributions

SP designed the study and acts as principal investigator. ALB was in charge of erythropoietin and biological management. SK was principal clinical investigator, SS, AB, ED, KB were clinicians and biologists involved in patients care. AD & OD supervised the study. All authors contributed to the interpretation of the analysis, read and approved the final manuscript.
